# Supplementary material for: Physicians’ perception of task sharing with non-physician health care workers in the management of uncomplicated hypertension in Nigeria: A mixed method study
Source: PLoS One. 2023 Sep 27;18(9):e0291541. doi: 10.1371/journal.pone.0291541 (PMC10529560; doi:10.1371/journal.pone.0291541)
Supplement: S1 File — (ZIP) [file pone.0291541.s002.zip › Themes and Sub-themes for the assessment of task sharing study---- report (1).pdf]

Project Partner Data Solutions
www.projectpartnerdatasolutions.com
+2348169518890; +2348073140024
ppdsife@gmail.com

QUALITATIVE STUDY REPORT ON TASK SHARING AMONG WITH NON-PHYSICIAN HEALTHWORKERS

PREPARED BY

Idowu Omisile

Contents

1. **Introduction** 3
2. **Doctors' Role in Patient Care** 3
3. **Effects of Shortage of Doctors on Hypertension Care** 3
4. **Effects of Non-Physicians without additional training in hypertension management** 5
5. **Benefits of Task Sharing** 7
   Benefits of Task Sharing to Patients 7
   Benefits of Task Sharing to Doctors. 9
6. **Impact of Task Sharing** 11
   Impact of Task Sharing on Medical Profession 11
   Impact of Task sharing on interprofessional rivalry 15
7. **Suggestions for Successful Task Sharing Implementation** 18
8. **Conclusion** 23
9. **APPENDIX** 24

1. Introduction

Indepth Interviews were carried out with twenty doctors (11 male and 9 female) in order to explore their perceptions on task sharing with non-physician healthcare workers in the management of uncomplicated hypertension in Nigeria. Several themes and sub-themes emerged from the discussions (Appendix 1). These are presented in the following sections and sub-sections.

2. Doctors' Role in Patient Care

At the outset, one of the interviewees identified two critical roles of doctors in the care of patients. The doctors were described as the first point of call in the care of patients and also as people who usually supervise the activities of non-physician health workers in the healthcare setting.

> *"Yes, the effect of the shortage of doctors on the management of hypertension is quite enormous and cannot be overemphasized considering the fact that doctors especially at the primary care level which is the first point of call, the doctors at this category, they are supposed to have a kind of supervisory role on other non-physician health workers" (Female)*

3. Effects of Shortage of Doctors on Hypertension Care

In all of the interviewees, the interviewees mentioned that the shortage of doctors has negative effect on hypertension care in Nigeria. In further exploration of their responses, specific impacts of shortage of doctors on hypertension care were mentioned to include increase in the patient to doctor ratio where we have more patients than doctors available; limited access to expert care; increase in waiting time for the patients; and increased workload for the doctors.

"Well the major effect is there are fewer doctors, there are more patients. Patients can't get across to their doctors, so there is obviously a problem with their diagnoses and management of patients that have hypertension, that's just the fact." (Male)

"The current shortage of doctors and health workers has generally placed much burden on the few doctors who are left, as well as the patients themselves. For instance. Where you have two or three physicians seeing maybe a 1000 of hypertensive patients before, and you now have one doing that. " (Male)

"Well the impact will be that patients will have limited access to expert care because most of the expert managers tends to be leaving so access to expert care will be limited and that

can worsen the hypertensive state of the individual who are been managed for such clinical condition" (Male)

"Also there are chances of those patients that are not having contact with professionals, I mean trained personnel's to have that can (1:33 inaudible) arise from hypertension and even, when they have access to it because of the long waiting time that they may have stayed, they may be discourage and not wanting to be compliance with their follow up clinic times." (Female)

"so even the qualified personnel's that are available they will be overwhelmed by the volume of patients they will be seeing in their clinics and also and the emergencies when they come down with complications." (Female)

As a consequence of the bottleneck in healthcare service availability, interviewees stated that the patients were likely to receive poor diagnosis and management of their cases, leading to increased complications in hypertension cases and increased death. Furthermore, patients were likely to be less compliant to treatment regimens and doctors would find themselves treating more complications instead of preventive practices.

"Well in so many ways it will have so many effects because number one, patients will not be well managed because there are not enough doctors to manage" (Female)

"It has really affected the management of hypertension in Nigeria because we really need doctors to achieve adequate control and management outcomes of hypertension in Nigeria." (Male)

", these few doctors available will not be able to attend to all the patients and some patients will seek for help elsewhere so by so doing, some of them may fall into the hands of quacks, people who are not doctors, who are not competent in the management of hypertension and it can have a disastrous effect on the patient because once they get to the place where things are not done properly, it could have a negative effect on the patient. You see patients poorly managed, you see patients coming down with complications which are not supposed to be there in the first place and ultimately could lead to the death of the patient. The effect is actually disastrous." (Male)

"We have more people dying from these complications because of the shortages but the primary management, I don't think there is so much change because other people have always been managing hypertension." (Male)

" In management of hypertension generally people, many patients will end up been non-compliance with both clinic visits and possibly with medications because, I mean the mass movement of doctors out of the country clinics are becoming crowded, over crowded people will be weary of staying in line to consult so it will have a deleterious effect on the management of hypertension generally and most specifically with compliance of clinic visits. " (Female)

"I think we're seeing the effect of shortage of doctors especially in tertiary health facilities now because we are more or less treating complications of high blood pressure like nephropathy,like heart failures. We're more or less treating complications rather than prevention of those complications." (Female)

4. Effects of Non-Physicians without additional training in hypertension management

The interviewees listed positive and negative impacts of having non-physicians without additional training engaging in hypertension management. In the positive view, the interviewees said having non-physicians working on hypertension management would lead to prompt management and treatment of hypertension as there would be more hands to attend to the patients. They further considered this to be beneficial because the non-physicians tended to be closer to patients compared to the physicians.

"It will actually help the populates to be better cared for. So, if non-physicians are properly trained and guided to manage hypertension, it's a chronic disease that have to be followed up for long. They can see somebody that will attend to them as at when due, during their clinic visits, somebody that can flag off complication or danger sign early enough. I think involving non-physician in the management of hypertension will make a positive impact because of the present lack of many specialists. " (Male)

"Presently today, our patients have access and also patronize them. There is nothing we can do about that because those non-physicians are nearer to the people. Today in our society, we have a lot of health care clinic being managed by non-doctors and people refer to them as doctors. Our system believed that any male person working in the hospital is a doctor. That is why if we don't train them, it may have a gross negative effect in the

management of this patients, and more patients will come down with more complication. " 13:3 ¶ 15, Presently today, our patients have access and also patronize them. The in D O

Several negatives outcomes were anticipated by the interviewees if non-physicians without additional training were involved in hypertension management. Generally, the interviewees opined that there would be poor management of patients and there was risk of death from this. Other anticipated challenges with having untrained non-physicians engage in hypertension management include that patients would develop complications, the non-physicians were most likely to miss critical disease indicators and they would give poor prescription and medication. They may also engage in unethical practices.

"That is a no brainer. It only means most patients will not get the adequate care they require. They will get half-baked management since they are not trained and skilled in management of hypertension. They are not knowledgeable in hypertension management " 2:1 ¶ 18, That is a no brainer. It only means most patients will not get the ade in PMO B

" I think I just have one which is probably they could overstep their boundaries, there is this tendency of them to managing complications and not knowing when to refer but I think if they have a guideline that clearly states when to refer" 3:2 ¶ 18, they could overstep their boundaries, there is this tendency of them t in R GWA

"Yeah, it could be disastrous because they will not know when to use additional drugs. They will not know the signs of maybe hypertension encephalopathy and They will not know when to send the patient to a doctor, because a regular doctor who has been treating a patient that has not been responding to drugs would send the doctor to the hypertension experts you know So, there will be more mortality and more strokes from such persons because part of the training will be when to send the patient to see a professional. " 19:2 ¶ 21, Yeah, it could be disastrous because they will not know when to use ad in P O

"definitely the patient will suffer because first of all this non-physician's health workers do not have the experience that the doctors managing hypertension have and of course it will lead to bad management again, increase complications, increase morbidity of patients " 14:2 ¶ 24 – 25, definitely the patient will suffer because first of all this non-physi in H K

" Death of the patient because the ultimate consequence is death of the patients. so, the key thing is the misuse of the medications and leading to bad outcomes. " 1:4 ¶ 20, Death of the patient because the ultimate consequence is death of the in C BENIN

"We face the challenges of poor prescription of hypertensive drugs, we face challenges of inadequate follow up by patient and non-compliance of routine checkups and medications and poor BP monitoring" 8:1 ¶ 18, We face the challenges of poor prescription of hypertensive drugs, we in R_Um

Apart from the risky treatment outcomes for the patients, the interviewees stated mentioned the possibility of systemic challenges in having non-physicians attend to patients. One of the interviewees mentioned that the non-physician health workers may begin to overstep their boundaries by engaging in activities that were not permissible to them. This was considered to be bad for the community and for the healthcare sector at large.

" I think I just have one which is probably they could overstep their boundaries, there is this tendency of them to managing complications and not knowing when to refer but I think if they have a guideline that clearly states when to refer" 3:2 ¶ 18, they could overstep their boundaries, there is this tendency of them t in R_GWA

"And to the community at large, in a particular community that is the highest thing that you treat and if the people that treat them they don't know their limits it can lead to bad image for the community," 11:12 ¶ 17 in O_Kwara

"The impact will be on the negative side because even those that are managing hypertension, I mean doctors are well trained for that have the (1:32 inaudible) having the knowledge of the metrics of action of the disease as well as the drugs to be giving for a particular stage of hypertension, of course hypertensions is more than been systemic, there are also other surgical causes so treating a patients with hypertension and leaving it in an unskilled hand, I don't think it will be benefit of the patient and even to the over all health care of the country eventually " 4:4 ¶ 17, The impact will be on the negative side because even those that are ma in R_IFE

5. Benefits of Task Sharing

Task sharing, as a critical topic of the study was evaluated in terms of the benefits it holds for patients and the benefits for doctors. These are discussed below:

Benefits of Task Sharing to Patients

The interviewees highlighted several benefits that would accrue to patients when there are non-physicians to augment the works of physicians in hypertension management. The most frequently cited benefits included that there would be reduced complications from hypertension, patients would enjoy closer relations with their caregivers (non-physicians) because they tend to live in the same communities, there would be easier access to care and reduced waiting time for the patients.

"The workload, one will be lesser for doctor and then complications that will follow undiagnosed or poorly monitored blood pressure will be lesser and will be relieved from the burden of having complications following hypertension." 8:3 ¶ 24, The workload, one will be lesser for doctor and then complications tha in R_Um

"Okay the benefits, the patients is going to actually help them have a good control of their hypertension, when these personnel are trained to manage this hypertension the patients will have good control." 5:5 ¶ 27, Okay the benefits, the patients is going to actually help them have a in R_K

"Also nearness of the non-physicians to patients where they live would also be an added advantage. Yes, these are benefits for the patients." 16:5 ¶ 22, Also nearness of the non-physicians to patients where they live would in M_LAG

"The people live longer they enjoy their life and then they will even have confidence in you because these people are the ones close to them, they can go to their homes and some other things like that they will even have more confidence in the care that is given to them based on that closeness so, complications is avoidable and they will have better care" 11:13 ¶ 21 in O_Kwara

"For those who have undergone some training It will reduce waiting time. Doctors are becoming extinct. It will reduce waiting time. We will have many hands that will be able to attend to those patients." 2:4 ¶ 21, For those who have undergone some training It will reduce waiting time in PMO_B

"awareness is going to increase, also it's going to reduce the queue we see at the general outpatient department mostly in the secondary and tertiary health facility. More or less we see those that are complicated." 3:8 ¶ 24, awareness is going to increase, also it's going to reduce the queue we in R_GWA

" It will improve access of patients to care and reduce the likelihood of complications. The non-physician health workers at the community are more likely know patients' language and where they live. They can speak. the language to them, they can interact with them. It's better for us, it's better" 13:4 ¶ 17, It will improve access of patients to care and reduce the likelihood o in D_O

"The people live longer they enjoy their life and then they will even have confidence in you because these people are the ones close to them, they can go to their homes and some

other things like that they will even have more confidence in the care that is given to them based on that closeness so, complications is avoidable and they will have better care" 11:13 ¶ 21 in O_Kwara

Apart from the above, the interviewees mentioned that involving the non-physicians would likely be less expensive for the patients and lead to much earlier diagnosis and treatment of hypertension. Furthermore, the patients would end up receiving the right information and medications as needed.

" *It will improve access of patients to care and reduce the likelihood of complications. The non-physician health workers at the community are more likely know patients' language and where they live. They can speak. the language to them, they can interact with them. It's better for us, it's better*" 13:4 ¶ 17, It will improve access of patients to care and reduce the likelihood o in D_O

"*I think the patient will benefit from that because those who have undergone training will be able to pick high blood pressure early, maybe from community diagnosis, by starting treatment on time, by advising them on treatment adherence, preventing complications.*" 3:4 ¶ 21, I think the patient will benefit from that because those who have unde in R_GWA

"*Apart from early diagnosis, starting treatment on time, those that are having elevated blood pressure,*" 3:5 ¶ 21, Apart from early diagnosis, starting treatment on time, those that are in R_GWA

"*So, the benefit of the patients are numerous. We are sure that they will get the right information. Some people don't like coming to the doctor for several reasons, religious beliefs and what have you. Going to them too, you're getting the right information, you're getting the right drugs.* " 16:3 ¶ 22, tant thing for everyone involved. So, the benefit of the patients are in M_LAG

Benefits of Task Sharing to Doctors.

On the part of the doctors, task sharing with non-physician healthcare workers was said to be beneficial as it helps to significantly reduce workload, while giving the doctors opportunity to play leadership/supervisory roles and other occupational priorities. Furthermore, it would lead to increased compliance and adherence of patients.

"the benefits are actually enormous because number one, the burden on the doctors will .actually reduce because this persons are now managed by this personnel's that have been ltrained so the burden on doctors will reduce because the number of patients that the doctors will have to see during clinic periods will be actually reduced that will also help the doctors to have more time for his or herself and also reduce the burden on doctors."

"Yes I think they will benefits in the sense that their work will be focused on those that need more attention, like those that already have End-organ Damage, their time will be focused on that so they will have less work load in the clinic, I mean the time they will need to attend to them they will dedicate it to platients that really needs their time"

"For one, the reduction in the workload, okay for one you can pass uncomplicated cases to non-physician then thereby relieving you of having to see them so you… gain more time so that they can see more complicated cases on time, and then relieve them of having to deal with a lot of patients"

"Yes, in the same arrangement, benefits of the doctors is reduced patients load and reduced redundancy because sometimes some doctors might feel that why am I wasting my time treating hypertension, is it not just check your blood pressure and move on. A doctor might feel like am not doing what I should do or wasting my time on this particular individual. So, for the doctor, one, it ensures that they see patients who are on a scale of preference who need their attention more than someone with uncomplicated hypertension. It ensures that the work load is lesser on them so they can focus more time, energy on people who are more serious health issues"

"Burnout will be reduced, the stress because you have passed the bulk to other people, so you'll be able to concentrate on things that really matter, by so doing it will reduce the burnout because the patient load will reduce, the mental task on you will be reduced because the simpler cases has been referred and delegated to other people who have been trained in it. So, for the doctor, it will reduce burnout, you will be able to also take care of other categories of patients that need expert care. I think the major issue or major benefits among others to the patients is this reduction in the burnout."

"Yes, patients will, if we're being honest. When they doctors are overwhelmed, they might not really pay attention to the patients; might just want to hurriedly see and move on. When we have these people that have been trained, we'll have situations where they can readily address this. Most non health workers like the feeling of someone coming with them with health issues, so they are better able to give their time and then we're sure the right information is being passed across which is the most important thing for everyone involved. So, the benefit of the patients are numerous."

"The benefits accruable to doctors, I think there would be eventually reduced work load for physicians generally, in that they get to manage cases that really cannot be managed by this non-physicians health workers and they can better manage the cases that come to them, other benefits, I cant think of any one right now."

6. Impact of Task Sharing

In another vein, the interviewees described the impacts of task sharing on the medical profession at large and on the inter-professional rivalry between physicians and other healthcare providers in particular.

Impact of Task Sharing on Medical Profession

The interviewees mentioned positive and negative impacts that task sharing on the medical profession. On the positive side, they said the act would lead to better management and monitoring of patients which has led to increased compliance of patients to treatments and reduced cost of care, which in turn improved the welfare of the patients' and reduced morbidity of the patients. Furthermore, the interviewees also said that task sharing have made the work system interesting and enjoyable for doctors and also brought about division of labour among the health workers.

On the negative side, the interviewees said it is risky for patient treatment, and it has permitted more quacks to practice, also led to non- physician's health workers usurping doctors.

"I think we (clears throat) have more of positive impact. For instance. When physicians have been trained formally, they get to see more patients, and then they can even pay home visits okay, and then look at compliance patients with their medications and so on and so forth."

“It will actually be beneficial to the profession because it will actually help to improve patient’s health and the like.”

“One, it will help work and it will help in early diagnosis, management, reduce the burden of hypertension and it’s complications in the community and health sector at large.”

“I think it makes treatment more effective, more effective. It will increase the number of people that have access to the right information concerning the management of hypertension and really, the results as well will be better.”

“It will improve interprofessional relationships because sometimes people tend to feel doctors try to be lords over them and all that. So having something like this will also Improve Interdependence among health workers, its good, it will improve relationship working relationship between doctors and other health workers definitely.”

“look there is no fear in this, even in America today we have nurses who are running some hospitals. You see I don’t know why we are afraid. I can't be afraid of anything once I play my role well and they play their role well. There is nothing wrong. It's only that you need a proper monitoring and evaluation system. When patients with hypertension are given long appointment such as three months, they can be referred to other smaller health care facilities that are close to them for regular BP monitoring by non-physicians health workers. There is no way proper mentoring can be done by only one doctor.”

“ No, the medical profession will not be watered down, instead I feel it will impact positively on the medical profession because ideally we have cadre of healthcare as people that are supposed to be seen at the primary care level, there are some people that are supposed to be seen at the secondary care level and at the tertiary care level. If the management too has been stratified so to say, it will make the medical practice interesting and enjoyable and even more effective.

“Of course, I mentioned quality care, quality care. Is a flow, the closeness of this health care workers with the patients is very very important because most of the time when you want to talk to those relatives when you send this people they believe them the impact will ensure quality care because everybody knows what they are supposed to do the next stage to move to so that by the time I have done my own part it moves to the next one so in all in

all accessibility this health care workers will have access to them in timing, they get health care on time before it gets to the next stage of complications"

"Yeah. I think it will improve medical profession because number one. There will be better working relationship no man is an island. No doctor is an island and when we think of our limited facilities and now shortage of doctors is better to bring more people into the space and the hypertension is rising because of so many factors today so we need more hands there are more hypertensive today so in terms of the medical profession."

"It will be a form of division of labour there will be a kind of shared responsibilities, so is going to make things easier in terms of doctors not been over worked, while other health care workers, just stay without doing anything so it will be like a kind of division of labour."

"It will make the medical practice more effective because medical practitioners or physicians who are working in the tertiary care level will be focused on what they are supposed to do, tertiary care or secondary care and they will not be bedeviled with other small small little little ailment which is supposed to go to the primary care facility. In my opinion it won't, in as much as they've been well trained, it.11:14 now everybody has their own limits you know when to refer, you know who to call, so the shortness of this access and other thing like that. It will help a lot even to gather data, that this people have contributed this and this, access to quality care in everything and this is one of the ultimate thing we want to achieve."

"So, the access for the patients is or to medical or to the non-physician to treat the hypertension is there. So, you have mm… by talking to them, by encouraging them you have an increase in compliance, which may reduce their burden"

"In my own opinion, on the long term, most other health worker will want to play doctors as we have in our country. It will give them the opportunity to start acting like doctors. In the long term, they may start challenging doctors. I don't think it is the way to go anyway"

"And then I think cost too generally, cost of seeing a physician, maybe consultation fee and all that too might reduce by a fraction."

“That is going to be a big one, a big one in the sense that we have a system where there is some degree of rivalry between the different cadre of the medical personnel so now bringing this unskilled individual into the picture to be managing disease condition like hypertension. I hope they won’t end up branding themselves as specialist doctors in managing hypertension simply because somebody in an attempt to make the work easier has empowered them in quote. so, there will be need for some kind of control to be put in place to avoid such events arising in the future.”

“ As it is now we have a lot of people who are practicing as quack and this may encourage more of that, and that’s why I said that it will require a lot of supervision and there will have to be a major of control, a major of check and balances so that people don’t just come up claiming that they have been trained and all of that.”

“if it is managed very well, something like Laboratory scientist, even nurses were all brought in to help the physician, but they are taking the, like antagonistic, like it's a different outlook now. That no, I am a professional of my own. This can go that way. If is not properly managed. If do's and don'ts are not set in place, if guidelines are not set in place, if things are not backed by let me go to say even law if they are not put in place then somebody can say we are the once managing hypertension all the while, so if that is not put in place it can go haywire. Like inter-cadre rivalry can come in but if it is properly managed like outside the (inaudible) people do their work o and there is no problem. For example A radiologist no a sonographer tis not a radiologist he does all the scanning but send it to a radiologist to report but in this Country if somebody is doing scanning he will think his a radiologist so my perception is if there are proper guidelines even to the extent putting it in writing I mean law they won’t go haywire but if there are no proper guidelines, just train you and you deploy they can take over and say they are the physicians. So that’s my fear.”

“You know they can be doing it privately even without, what I mean is that they will even be doing it privately without doctors supervision, they will think, they will have this believe that they are now very okay to manage such conditions and also this may lead to… they might take over doctors job and the masses might not really know the real difference

between a medical doctor and other health care providers as far as they are seen to be treating their cases."

"The impact will be on the negative side. There is nothing positive about it. One, if they are managing uncomplicated hypertension, believe you me,it will soon go to complicated because they don't know the ethics. They don't know anything in as much as they have been trained but they are not qualified to do so. So in my opinion, I don't even accept that, I don't believe that. It should be managed by medical professionals."

Impact of Task sharing on interprofessional rivalry

The impact of task sharing on interprofessional rivalry as deduced from the study were included increased rivalry among other health professionals because the non-physician healthcare workers were likely to be pushing a sense of entitlement, which could also lead to an escalation of patients' problems. However, it was said that it could also positively impact interprofessional rivalry because of better relationship among the healthcare workers and also leading to positive outcomes for the patients.

"But on the other hand, if you look at it, it might get out of hand. Because for example, if you look at what they do in all these road side pharmacies these days they treat patients, they do a lot of unto worth things so then it might go that way because they feel they know about hypertension, have been trained so. So sometimes when it gets out of hand and they're supposed to actually refer to a physician, they may keep managing the patients, looking for various ways of bringing down the BP which might actually go out of hand So, in these ways, that's why I think the rivalry might increase."

"I think it might be more of negative than positive. Because if we're being honest, one of the causes of inter professional rivalry is insecurity. We have individuals who are insecure in their roles, who are insecure in what they are doing, who feels I'm a bit better that this person, all of that. If that has not been addressed from the baseline, you now further teaching them or giving them more information that doctor has, not like information should be hoarded, now make them feel like there is nothing you know I don't know; there is nothing special about what you're doing. On my own part, I will be happy sharing my knowledge, I mean knowledge should not be hoarded, the most important thing we need to consider is the patient."

This one seems to be a difficult one to answer. Ideally, it’s not supposed to have any negative impact. We should see ourselves working together as colleagues and complimenting one another but with the present situation which is quite unfortunate in the health sector it may have some negative impact because some people will feel that they are now in charge. Now they have gotten training that the doctors has so they can now manage hypertension, they will be in charge, some will take the responsibility of a doctor and they won’t know their limits. They will tell the patient’s, I’m also doctor so so so and so and I can treat you. So with this ego in the profession between the doctors and nondoctors, somehow indirectly it have negative impact because this people want to hold on to the patients and do what the doctors are doing. So, I’m that respect, it may bring an unhealthy kind of relationship which is not supposed to be.”

“I think the fear should come probably if it’s a procedure, that’s when the doctors will be kind of apprehensive probably, they will displace us but this is not a procedure. I think it should be incorporated as part of non-communicable diseases that other healthcare workers should manage.”

“I think to some extent it will make relationship with the doctors better, and doctors will be showing leadership in the health team. We are…Leadership has been entrusted on doctors and it's our responsibility to look at the allied health professionals and bring them in. So, I think things will work better and doctors will be viewed more favorably as leaders and initiators of change for the better in the health sector.”

“Well I think is going to have a positive effect because some of this non-physician health workers actual some of them actually feel that doctors claim that, they are more superior and so they should manage all cases and the like so is going to actually help to promote good relationship, interpersonal relationship and the likes.”

“It will improve interprofessional relationships because sometimes people tend to feel doctors try to be lords over them and all that. So, having something like this will also Improve Interdependence among health workers, its good, it will improve relationship working relationship between doctors and other health workers definitely.”

“It will not be a good one because, normally they’ve been trying to rub shoulders with medical doctors and if they are allowed to do such, it will bring more harm than good because they will now feel that they are qualified to treat anybody in question, you know?. They will want to over do their thing. From uncomplicated hypertension, they may even go over to complicated, thereby worsening the issue because they don’t have any background knowledge about it.”

“but the bad is even before now we have non-physicians trying to take over the role of doctors, trying to take over the role of the physicians, trying to feel like a physician, so when we now have this, if stringent laws are not put in place or boundary lines are not properly drawn, we would have these people even further taking over these roles of the physicians. An average patient out there doesn’t know the difference, so any non- physician health worker who has been trained can easily pose as a doctor with confidence and the patient will believe and so if they don’t also know where to draw the line, they will go ahead and keep treating and not know when to refer or not know when to further escalate upwards.”

“Okay, I don’t think this is a kind of procedure, already the non-doctors have been taught to manage uncomplicated conditions like malaria, diarrhea, other diseases that are not severe, I think incorporating management of uncomplicated hypertension wouldn’t be different from that, because they know when to refer these conditions, like when they have severe malaria they know when to refer, when they have severe diarrhea disease they know when to refer, like when the patient has severe dehydration they know when to refer. I don’t think this would be different.”

“If you don’t mind me saying, I wrote a paper on inter-cadre rivalry in the health care system is multifactorial and if you do your research you will find out it is remunerations and welfare packages and not necessarily professional competence. Every health care profession have their job schedules and descriptions base on their training so it will be odd for a pharmacist for example to insist on performing appendectomy, have never know that happen before neither will a med lab scientist go ahead to try and do an emergency caesarean section because he feels he is equal to a physicians.I think the issue of inter cadre rivalry as to do with remunerations and welfare packages so let’s be sure we are

separating the two. So as regard clinical competence as regard clinical acumen as regard what clinical responsibilities I don't think inter cadre rivalry as such major impact on it, so I therefore say as far as task sharing is concerned its less likely to have issues with inter cadre rivalry, I know so because nurses and pharmacist and med lab scientist who pray that their children will become doctors and that has always been a very interesting perspective for me and as the same time, they feel that doctors are gaining too much from the system. You cannot eat your cake and have it so the principle of task sharing is not likely to worsen or have much of an impact on inter cadre rivalry if we focus on the protocols if we focus…. on during the training on the limitations required, and from my experience the training will help the non-physicians to know his or her boundaries."

"But if things are placed in proper perspective if there are task sharing guidelines, this is what you can do, this is what you cannot do. If it's placed on and people respect it in Nigeria, if people respect it there will not be any problem. But if people will not respect it and they cross boundary it might worsen the inter-cadre rivalry that is still on presently in the country."

Well under supervision is fine is a way to curtail such rivalry but I think the supervision should go beyond just ordinary supervision to have a well documented documents with respect to policies that will guide such individuals, such that once they are stepping out of the policy or out of the document that guides their practice they can be called to stay at the right path".

7. Suggestions for Successful Task Sharing Implementation

Therefore, to successfully implement task sharing, it was strongly recommended that the non-physician health workers should be properly trained and supervised, and that their job should be effectively described and communicated as this will would help clear any ambiguities. Furthermore, it was recommended that there should be a limit on the scope of non-physician healthcare workers' scope of authority in patient care and that if possible, only a selected number of them should be trained and allowed to engage in hypertension management. Furthermore, it was proposed that there should be deployment of effective control mechanisms, establishment of policy monitoring team and creation of a well-designed algorithm and a friendly environment.

“It may have and it may not have but of course you are dealing with human beings here so you cannot outrightly say that there won’t be. But the only thing to do is to guide against it, you cannot actually say that it will not, it may because everybody wants to be a doctor either by crook or by any path available.”

“Well it can actually be successfully implemented by setting up committees, set up people for adequate planning, is actually very important and likewise good training for those that will be involved. And the committees will help.”

“Then secondly there should be a very good environment, the environment should be a friendly environment, it should not be a place that people are working and they are not happy, definitely they will get on other people’s nerves and it will not be good for relationship, then there can also be time to time seminars for the health care professionals together not segregating them, for them to learn good working relationship, that way it will help benefits them and the patients too.”

“and that’s why I said that it will require a lot of supervision and there will have to be a major of control, a major of check and balances so that people don’t just come up claiming that they have been trained and all of that.”

“If that inter professional relationship is okay which comes with good communication, the doctors should not always play god, sometimes the doctors should always come down from that high pedestal and also make these people feel they are important. We should make them feel that they are also important, as doctors and physicians, physicians should make them feel that this is a complementary work and we cannot do it alone. We should know that this Is a team work and we cannot do it alone , make them understand their relevance and their place in the overall healthcare service delivery. So once that is done, you get them by your side and everything will work well”

“ One of the way the negatives can be minimized on the aspect of the health workers is by always reinforcing the importance of every person, knowing that irrespective of whatever title you bear, you’re important in this and that not everyone can be a doctor, not everyone can be a nurse. If we have all doctors, who will be the nurse. Everybody should know that wherever you find yourself you’re important, so that should be reinforced and then also

when we're task sharing let things be properly defined and spelt out so that everyone knows what is expected of them, just like what we have in PHC where we have things like this diarrhea, you know what to do and when to refer Things should be clearly defined and spelt out"

"Effective job description, if you are in a team of management of uncomplicated hypertension, let every body know their job description and if you an attendant too, when you are to do this thing. Everybody know their job description that as a nurse by so so time you can give some injection, they don't go beyond their boundary, so a well outline job description will help and they know that if you step out of your boundary, you do things that you are not suppose to do, you are liable to punishment because of one misconduct or the other."

"But like I said and keep on saying, a good referral system is lacking in Nigeria today. If we refer people to the other physicians, they should be able to tell us what they have done and refer the patient back and inform us about next line of management. When we have a protocol and have a proper monitoring and evaluation. It will be best for our systems. So, we won't see ourselves as enemies. We see ourselves that we want to achieve a goal. And the success of this program is for all of us. But the point is that the person who is sick needs to be helped and if we have a team, we can achieve more but a single person cannot really achieve anything. And I believe that is how it will work"

"My suggestion is that there should be a team that sees to that, it should not just be open to. There should be a team in per centre, I mean it could be per state it could be per region that sees to the control of this implementation of this policy, like a group of people that they are will be reporting to"

"if they have a guideline that clearly states when to refer I think it will go a long way in preventing complications of hypertension."

but most importantly I think the training should also come with an accompanied policy in a document form that will guide their actions, their practice per time so that they do not go beyond the limit or the confined of that practice and they should know when to call in the expert for cases that are beyond their control. The training is fine but beyond that, a

documented policy must be in place to guide the practice. “For you the advice will be you need to be strict about putting down protocols for the management based on the patients’ blood pressure level, what test they could do what drugs they could give and so on and let them know that beyond this point they have to refer to a physician. And I think that is the way to go. Just make sure everything is down on paper everybody must know their job description and if you make the mistake of increasing the remuneration of your non-physician health workers who will be treating uncomplicated hypertension without considering the physician that is doing the supervision that physician may protest so be ready to spread the remuneration so that one will not feel as if he has been left out on this welfare package. So, that is my advice.”

“We should train them and let them know their limitation. They can use algorithm in patient care that tells them which group of patient they can manage and not manage; when to refer; who to refer to or the superior to call next. This has been used with positive results in obstetrics care in Ondo State We can now have protocols. Then the doctors, nurses, the pharmacist we should be able to sit down and design a protocol of management for hypertensions and boundaries should be set in terms of when to refer.”

“What you are doing now is number one. Get the opinions of the health workers; not only the doctors but other health workers such as pharmacists, nurses, lab scientist because in Nigeria. We should also seek the opinions of the unions because in Nigeria, you can’t run away from unions like the Nigeria Medical Association, Medical and Dental Consultants of Nigeria, the Nurses Union. Let them be well represented to know that this is a problem at hand, how do we do it? If we can call for a conference, we can send representative, let us talk together let's talk to ourselves, this job is too overwhelming, you can't do it alone. We want you to also be around to help us to practice. If you are saying that is only doctors will take the BP, do the echo, do that, ah ah it is burdensome.”

“And basically, I think my suggestion would be that people are well exposed. Like I said, make it available to health workers and non-doctors. And there should be…there should be enough publicity given to it. And to ensure that people are also well trained before they start embarking on that. And maybe there should be a way of certifying that people have the requisite knowledge before giving them something like to check, before allowing them

to go ahead and participate in that. They should be able to check. they should be able to properly train and then to now check after the training to ensure that they know what they're supposed to know. They have good knowledge or good grasps of what they're supposed to do before allowing them to do that. So, I think if that is done its good and they should also be well monitored."

"So, there should be a structure probably a Telemedical System that allows the physician from his advantage point to be able to observe what is going on even though he or she is not present physically. But the key thing is supervision. So ultimately the doctor will still be in charge, must be in charge of the care. So, there shouldn't be any time where people will now say it is non-physician health worker that is in charge. That level of responsibility will probably give us better result."

"And there should be a major of check and balances on those who have been trained so that they do not start practicing outside of supervision which will make things worse for the people they are managing and the masses that trust them already. So, checks and balances, really in the practice and then a control, a sort of control in the procurement of medications so that people procure medications following prescriptions, appropriate prescriptions from certified health workers even if they are non-physicians, rather than just go to any shop and say they ask me to get this and all of that. so, checks and balances. A system that controls and guides the non-physician's health workers to appropriate management and also a guideline a protocol as it where to recognize patients that needs to be referred, protocols that details the red flags, that dictates who need to be referred and who they can manage. I think if these are put in place it will be a good one."

"my suggestions would be that we should not let it be open for every healthcare worker okay. We can select some few maybe the pharmacists, the nurses, and then probably some senior community health extension workers okay. Maybe they should put a limit to those to be trained, maybe the senior ones amongst them who are more experienced."

" well designed algorithms that are not ambiguous, that are easy to follow and then number three very good referral system, so that when a patient is referred to see health care professional, his giving the necessary attention promptly, because already that patient has been screened."

8. Conclusion

Task sharing poses great benefits to the medical profession and patients. It reduces workload amongst the doctors and reduces morbidity and complications. However, it could escalate problems with NPHW and increase interprofessional rivalry amidst health workers. In conclusion, it was recommended that there should be proper training and a round table discussion as this will encourage teamwork.

9. APPENDIX

THEMES AND SUB-THEMES

Themes	Sub-themes
00 Sociodemographics_Profession	doctor
00 Sociodemographics_Sex	female
	male
01 Effects of Shortage of Doctors on Hypertension Care	○ increased disease complications
	○ increased patient-doctor ratio
	○ poor diagnosis and mgt
	○ increased workload
	○ increased waiting time
	○ non-physicians treating patients
	○ increased deaths
	○ limited access to expert care
	○ patients non-compliance
	○ negative effect
	○ more complications less preventions
02 Effects of Non-Physicians without additional training in hypertension mgt treating hypertension	○ poor management of patients
	○ Death
	○ patients develop complications
	○ unethical practices
	○ Positive impact
	○ poor prescription and medication
	○ NPs miss critical disease indicators
	○ NPs closer to patients
	○ patients spend more
	○ negative effect
	○ NPHW overstepping boundaries
	○ not in patients' best interest.
	○ bad for healthcare sector

	○ poor patients' compliance
	○ bad image for community
	○ prompt management of HTN
l03 Benefits of Task Sharing to Patients	○ reduced complications
	○ closer relations to non-physicians
	○ reduced waiting time
	○ easier access to healthcare
	○ early healthcare access
	○ higher community-level awareness
	○ less expensive
	○ early diagnosis of HTN
	○ getting right drugs
	○ get right information
	○ early commencement of treatment
04 Benefits of Task Sharing to Doctors	○ Reduced workload
	○ higher compliance and adherence
	○ opportunity for leadership/supervisory roles
	● chance for other priorities
l05 Impact of Task Sharing on Medical Profession	○ NPHW trying to usurp doctors
	○ division of labour
	○ beneficial impact
	○ better management of patients
	○ increased rivalry
	○ better monitoring of patients
	○ reduced morbidity from HTN
	○ increased compliance with treatments
	○ better work system for doctors
	○ interesting and enjoyable
	○ improvement of patients' welfare
	○ reduced cost of care

	○ more quacks enter practice
	○ risky for patient treatment
06 Impact of Task sharing on interprofessional rivalry	○ could increase rivalry
	○ NPHW trying to usurp responsibilities
	○ No negative impact
	○ Negative impact
	○ positive
	○ enhanced relationship
	○ could escalate problems with NPHW
	○ entitlement
	● positive for patients
07 Suggestions for Successful Task Sharing Implementation	○ Supervision of NPHWs
	○ establishment of guideline policy
	○ proper training of NPHWs
	○ effective job description
	○ efficient communication
	○ train only selected personnel
	○ deploy effective control mechanisms
	○ establish policy monitoring team
	○ creating friendly environment
	○ adequate planning
	○ well deisgned algorithm
	○ Doctors should be accommodating
	○ limit scope of NPHWs authorisation
	○ inter-professional roundtable should hold
	○ improved referral system
	○ encourage team work
