# Supplementary material for: Physicians’ perception of task sharing with non-physician health care workers in the management of uncomplicated hypertension in Nigeria: A mixed method study
Source: PLoS One. 2023 Sep 27;18(9):e0291541. doi: 10.1371/journal.pone.0291541 (PMC10529560; doi:10.1371/journal.pone.0291541)
Supplement: Attachment — Submitted filename: RESPONSE TO REVIEWERS.docx [file pone.0291541.s003.docx]

We appreciate the comments and suggestions received from the reviewers towards improving the quality of our manuscript.

|  | REVIEWERS’ COMMENTS | AUTHORS’ COMMENTS |
| --- | --- | --- |
| 1 | My only suggestion is to check some typos in the tables and in some parts of the text. It seems that some symbols were missing | This has been done |
| 2 | However, I noticed quite a few grammatical and typographical errors that may detract from the overall quality of the work. These affect the clarity and coherence of your arguments and distract readers from the substance of the research. I believe these should be corrected. | This has been done |
| 3 | In addition, usually Task shifting and Task Sharing acronym is TSTS or TS/S. Whydid the authors use TSSH? | This has been changed to TSTS |
| 4 | The team mentioned 1250 doctors interviewed but the sample size mentioned in the methods section was much lower, please explain. | The study was a mixed method study. 1250 doctors participated in the online survey(quantitative part) while 20 doctors were interviewed for the qualitative part of the study |
| 5 | The Likert scale utilized in the study identified a score of 42 and above as indicative of a positive perception, as it was the mean value of the dataset. However, it is suggested that the scale could have been partitioned into three categories rather than two, with positive perception, negative perception and neutral perception based on the distribution of scores for more detailed analysis rather than classifying good and bad just by a point difference between them on a scale | Thank you once again for this suggestion. We opted to use the positive and negative perception for a concise discussion while we acknowledge the argument about using midpoints opinions on the Likert scale and the epistemological connotation of such.  However, the analysis decision was based on suggestions from previous literature on analyzing Likert items and the Likert scale amidst many arguments and what we felt was best in the context of the objective of the study.^[1],^^[2],^^[3],^^[4]^  We combined the items in order to generate a composite score (Likert scale) of the 12 items for the different participants, this thus enabled us to turn the assigned scale into an interval scale which we partitioned into two because we felt it was more beneficial to report the perception of the physician as positive or negative as that has significant implication for care and programme planning considering the importance of the topic rather than reporting as an ordinal variable with the midpoint opinions.  We agree that the scale could be partitioned in the manner suggested; however, as said earlier we decided based on the abovementioned facts. In addition, we ensured we created Likert scale items by calculating a composite score (sum and mean) from the 12 Likert items so we can use parametric statistics such as mean for central tendency and standard deviation for variance in keeping with research based on Likert items and scales in previous literature that treats them as interval scales and analyzes them as such with descriptive statistics like means, standard deviations, etc. and inferential statistics.  Sullivan GM, Artino AR Jr. Analyzing and Interpreting Data from Likert-Type Scales. J of Grad Med Educ 2013DOI: <http://dx.doi.org/10.4300/JGME-5-4-18>  ^2^ Subedi BP.Using Likert Type Data in Social Science Research: Confusion, Issues and Challenges. Inter J Con App Sci. 2016:3(2). www.ijcas.net  ^3^ Rickards G, Magee C, Artino AR Jr. You can’t fix by analysis what you’ve spoiled by design: developing survey instruments and collecting validity evidence. J Grad Med Educ. 2012;4(4):407–41  ^4^ Draeger, R. Cut-off scores. In M. Allen (Ed.), The sage encyclopaedia of communication research methods 2017: pp. 326-328. SAGE Publications, Inc, https://dx.doi.org/10.4135/9781483381411.n122 |
|  |  |  |
| 6 | The Results sections should be rewritten as it is quite verbose with long sentences by the respondents which do not add to the analysis and could be summarized under the themes with some more analysis in the section. The tables could also do with some re-arrangement for ease. | This has been revised as suggested |
| 7 | Under the discussion section, please explain what you mean by brain drain and quacks to assist with clarity, in addition to some other terms in the text for an international audience. This is especially important to remove any suggestions of inherent bias since majority of the authors are doctors. In addition, distinguishing health trained versus non health trained staff, and which health workers can carry out management of hypertension in low resource settings such as where the study took place compared to tertiary health care settings could do with a literature check | Brain drain has been replaced  Quack has been replaced  The emphasis of our study was non-physician health workers |
| 8 | Line 341-The authors say *Task sharing is therefore, likely to address the consequences of poor patient-physician relationship*. This statement will benefit from a reference, or it should be reviewed | This has been reviewed.  The statement has been expunged |
| 9 | The Conclusions and recommendations (381-386) need to be reviewed completely as recommendations based on interviewees perceptions are quite different from author recommendations based on scientific enquiry and findings as well as the literature review. The section requires a complete rewrite and needs to be organised. | The conclusion and recommendation have been revised as suggested |
| 10 | There are a number of references of work done on hypertension in Nigeria recently that should be reviewed and considered to improve the document, especially with relation to lower cadre health workers in the area of hypertension. | More literature from Nigeria has been added |
| 11 | The Nigerian TSTS guidelines have been reviewed and the changes should be included in the paper with relation to various cadres of health workers in the literature review section so that the article is not outdated even prior to publishing. | Thank you for this observation and suggestion.  We have provided more information on TSTS policy on communicable  However, on further enquiry we found that the TSTS policy document on non-communicable disease is yet to be published officially. It is being considered administratively at the Federal Ministry of Health |
