# Supplementary material for: Physicians’ perception of task sharing with non-physician health care workers in the management of uncomplicated hypertension in Nigeria: A mixed method study
Source: PLoS One. 2023 Sep 27;18(9):e0291541. doi: 10.1371/journal.pone.0291541 (PMC10529560; doi:10.1371/journal.pone.0291541)
Supplement: Attachment — Submitted filename: PONE-D-22-34586R1_Comments.docx [file pone.0291541.s004.docx]

**Physicians’ Perception of Task Sharing with Non-Physician Health Care Workers in the**

**Management of Uncomplicated Hypertension in Nigeria: a mixed method study**

**Introduction**

1. Line 83-84: *The World Health Organization’s (WHO) report showed that hypertension affects about 1.3 billion adults worldwide* – **this statement needs to be cited.**
2. Line 95: The word **‘brain drain’** still appears (despite previous assertions that it was replaced). Is this intentional?

**Methods**

1. Satisfactory

**Results**

1. Line 197: Clarify whether the 1250 were for just the quantitative aspect of the study or for both qualitative and quantitative.
2. Line 202 -203: A significant proportion (43.4%) have a negative perception about TSTS. This will suggest that the support is not overwhelming. Their concerns need to be explored to identify what points needs to be improved on.
3. Line 204 -208: This needs to be clarified; the percentages of those who believe trained non-physician HCWs need or do not need supervision is at variance/ confusing.
4. Line 208 -212: It will be beneficial to also state the percentages here.
   Also, **was the level of comprehension/ understanding of the TSTS concept** among the respondents assessed? It will have been useful to compare their **level of understanding of the concept** with their **perception.** Because, if they had a good understanding of the concepts, and still did not give an enthusiastic endorsement, then there are clear concerns.
5. In addition, it will have been beneficial to narrow the respondents of this study to those who are involved, in some capacity, in the treatment of patients with hypertension.
6. Line 213 -222: These parameters used for the analysis here are rather generic for the evaluation of such an important policy.
   i. What proportion of physicians recruited in this study are involved in clinical work?
   ii. It would have been more informative if the inclusion criteria for this study had been better defined at the beginning.

**Discussion**

1. The discussion highlights the findings of positive perception among the respondents, without addressing the reasons for the significant proportion of respondents with negative impression.

In order to get the best out of this policy, the concerns of those with negative perception will need to be highlighted and addressed.

2. Also, it is worthy to note that the loss of HCWs in the country is not just among physicians, but other professionals cadres as well. **What cadre are the authors proposal in this roll-out?**
