# Supplementary material for: Physicians’ perception of task sharing with non-physician health care workers in the management of uncomplicated hypertension in Nigeria: A mixed method study
Source: PLoS One. 2023 Sep 27;18(9):e0291541. doi: 10.1371/journal.pone.0291541 (PMC10529560; doi:10.1371/journal.pone.0291541)
Supplement: Attachment — Submitted filename: RESPONSE TO REVIEWERSp.docx [file pone.0291541.s005.docx]

|  | REVIEWERS’ COMMENTS | AUTHORS’ RESPONSE |
| --- | --- | --- |
| 1 | Line 83-84: The World Health Organization’s (WHO) report showed that hypertension affects about  1.3 billion adults worldwide – this statement needs to be cited. | Reference added |
| 2 | Line 95: The word ‘brain drain’ still appears (despite previous assertions that it was replaced). Is  this intentional? | Brain drain has been deleted |
| 3 | Line 197: Clarify whether the 1250 were for just the quantitative aspect of the study or for both  qualitative and quantitative.  . | 1250 was for quantitative. This has been clarified in the method |
| 4 | Line 202 -203: A significant proportion (43.4%) have a negative perception about TSTS. This will  suggest that the support is not overwhelming. Their concerns need to be explored to identify what  points needs to be improved on | In the study, we explored various concerns, including the one mentioned above, through the qualitative component of the study. It's important to note that perception can vary depending on the context, which is why we conducted the qualitative assessment. The qualitative component sheds light on this.The Theme number 5 of our qualitative results identified some concerns raised by some physicians which include promoting inter-professional rivalry and encourage unprofessional conduct among lower cadre health staff  This has also be discussed |
| 5 | Line 204 -208: This needs to be clarified; the percentages of those who believe trained non-  physician HCWs need or do not need supervision is at variance/ confusing. | This has been clarified |
| 6 | Line 208 -212: It will be beneficial to also state the percentages here.  5 | This has been clarified. |
| 7 | Also, was the level of comprehension/ understanding of the TSTS concept among the  respondents assessed? It will have been useful to compare their level of understanding of the  concept with their perception. Because, if they had a good understanding of the concepts, and  still did not give an enthusiastic endorsement, then there are clear concerns. | No, it was not assessed. The concept of TSTS was defined and introduced to the respondents before they started filling the question  We agree that their understanding could have been tested and be related with their perception. This has been included as limitation of the study |
| 8 | . In addition, it will have been beneficial to narrow the respondents of this study to those who are  involved, in some capacity, in the treatment of patients with hypertension. | Comments well noted  All consenting physicians were captured, however, majority(88%) were involved in clinical practice. The perception of physicians who were not directly involved in hypertension management such as those who are involved administration and policy making may also be important for successful implementation of TSTS Strategy |
| 9 | 6. Line 213 -222: These parameters used for the analysis here are rather generic for the evaluation  of such an important policy. | The mixed method was aimed at addressing this concern, like said earlier in question1. The usage of bivariate analysis established existence of association, and the qualitative component idea was to attend to the contextual nature of the research idea rather than quantitative assessment only. |
| 10 | What proportion of physicians recruited in this study are involved in clinical work? | 1100 (88%) of the physician were in clinical work at the time of the study. It’s presented in line 508 -table . |
| 11 | It would have been more informative if the inclusion criteria for this study had been better  defined at the beginning. | Now included.  Only licensed and practicing physicians who were either previously or currently involved clinical work were included in the study. |
| 12 | The discussion highlights the findings of positive perception among the respondents, without  addressing the reasons for the significant proportion of respondents with negative impression.  In order to get the best out of this policy, the concerns of those with negative perception will need  to be highlighted and addressed. | This has been addressed in Page 10, Line 335 to 343 |
| 13 | . Also, it is worthy to note that the loss of HCWs in the country is not just among physicians, but  other professionals cadres as well. | Yes, we agree that migration of HCWs out of the country affects non-physicians health workers. However, our study is focused on the Physicians because they are the ones whose role in hypertension management will be shifted and/or shared |
| 14 | What cadre are the authors proposal in this roll-out? | The plan is to assess the perception of non-physician health workers about TSTS. The findings will be useful to make useful recommendations that will facilitate the successful implementation of this policy |
